# Identification of Parasite-Host Habitats in Anxiang County, Hunan Province, China Based on Multi-Temporal China-Brazil Earth Resources Satellite (CBERS) Images

**DOI:** 10.1371/journal.pone.0069447

**Published:** 2013-07-29

**Authors:** Zhijie Zhang, Robert Bergquist, Dongmei Chen, Baodong Yao, Zengliang Wang, Jie Gao, Qingwu Jiang

**Affiliations:** 1 Department of Epidemiology and Biostatistics, School of Public Health, Fudan University, Shanghai, People’s Republic of China; 2 Key Laboratory of Public Health Safety, Ministry of Education, Shanghai, People’s Republic of China; 3 Laboratory for Spatial Analysis and Modeling, School of Public Health, Fudan University, Shanghai, People’s Republic of China; 4 Ingerod, Brastad, Sweden; 5 Laboratory of Geographic Information and Spatial Analysis, Department of Geography, Faculty of Arts and Science, Queen’s University, Kingston, Ontario, Canada; Centro de Pesquisa Rene Rachou/Fundação Oswaldo Cruz (Fiocruz-Minas), Brazil

## Abstract

Remote sensing is a promising technique for monitoring the distribution and dynamics of various vector-borne diseases. In this study, we used the multi-temporal CBERS images, obtained free of charge, to predict the habitats of the snail *Oncomelania hupensis*, the sole intermediate host of schistosomiasis japonica, a snail-borne parasitic disease of considerable public health in China. Areas of suitable snail habitats were identified based on the normalized difference vegetation index (NDVI) and the normalized difference water index (NDWI), and the predictive model was tested against sites (snails present or absent) that were surveyed directly for *O. hupensis*. The model performed well (sensitivity and specificity were 63.64% and 78.09%, respectively), and with further development, we may provide an accurate, inexpensive tool for the broad-scale monitoring and control of schistosomiasis, and other similar vector-borne diseases.

## Introduction

Schistosomiasis japonica, a snail-borne parasitic disease of considerable public health and economic significance [1]–[4], has existed in China for over 2000 years [5]–[9]. The Chinese government initiated a national control program to combat this disease soon after the revolution in 1949, resulting in substantial progress in the following 60+ years [5], [6], [10]. The numbers were reduced with more than 90% of the peak estimates between 11.60 and 11.85 million infected people [11], [12]. According to the national schistosomiasis report of 2010, the cases of *S*. japonica infection and the people at risk were estimated to be about 325,824 and 68,536,200, respectively [11]. Despite this success, there is considerable concern that schistosomiasis might re-emerge as active transmission has been frequently reported in areas that previously reached the criteria of transmission interruption or transmission control [6], [12]. There are several possible reasons for this: Firstly, habitats of *Oncomelania hupensis*, the intermediate snail host, are still widely present and this poses a strong risk for the reemergence of schistosomiasis in China [13], [14]; Secondly, the compliance rate for repeated drug treatment, the major component of the World Bank Loan Project (WBLP) strategy for schistosomiasis control, has declined substantially [15], [16]; Thirdly, reduced financial resources for schistosomiasis control make it hard to maintain the scale of chemotherapy and environmental modification at their previous levels [4], [7], [17]; Finally, the distribution of *O.hupensis* in China is restricted to the country’s southern parts, where its distribution is strongly governed by the temperature at the macro scale [13], [18], [19]. In the North, because of the climate change experienced since the mid 1900s, the temperature is now several degrees higher, which may eventually result in shifting of snail habitats northwards [19], [20]. Besides, the construction of the huge hydraulic projects such as the Three Gorges Dam and the South-to-North Water Transfer Project will also have a large impact on the local ecology, which will work in union with the ongoing climate change, possibly resulting in substantial new areas becoming suitable habitats for *O.hupensis* [21]–[23]. Hence, it has been suggested that an early warning system (EWS) should be set up in China to monitor the changes of distribution of the intermediate host snail habitats [10].

We know that *O. hupensis* is the sole intermediate host of *S. japonicum* in China and its distribution largely governs the distribution of *S. japonicum* [13], [24], [25]. Hence, understanding the distribution of snail habitats is vital for controlling this disease. However, identifying snail habitats requires considerable manpower and some areas are difficult to access, especially during flooding [26], [27]. Traditionally, direct surveillance of snail habitats (field investigation) in historically endemic areas and suspected new areas has been widely used [4], [28], [29]. However, this approach requires considerable manpower and is often not feasible in areas difficult to access [26]. In addition, snails can be difficult to spot because of small size (less than 1 cm) and because their color resembles that of the soil [30]. As is well known, the survival and reproduction of snails are closely related to environmental factors, water and vegetation being the most important ones, a fact that makes them possible to be traced by remote sensing (RS). Geographical information systems (GIS), backed by RS images, can be used to identify suspected snail habitats based on the special ecological characteristic of snail habitats that can be summarized as “land in winter – water in summer” and “no grass – no snails” [26], [27], [31]–[36]. However, most previous schistosomiasis studies using RS to detect snail habitats only selected two images, one from the dry season and one from the wet season to (a) first locate the water regions in each image either using the (un)supervised classification approach [26] or the tasseled-cap transformed wetness index [34] and (b) then identify the differenced water regions through the subtraction algorithm. After using the normalized difference vegetation index (NDVI) to derive the regions with vegetation coverage in the dry season, the potential snail habitats would be found in the overlapping areas. Although valuable, these studies have several potential limitations:

1. The selection of the two RS images from the wet and the dry seasons is always subjective, requiring familiarity with the local conditions to make the correct choice. Thus, this method is not appropriate for areas with which researchers are not well acquainted. In addition, the right dates for the dry and wet seasons are not easy to determine;
2. The threshold of NDVI for detecting the vegetation coverage is subjective and different values are used (e.g., 0 [34], 0.1 [37], 0.2 [38]);
3. The approaches to identify the aquatic regions are numerous and require users to master certain RS techniques;
4. The Enhanced Thematic Mapper (ETM) is the most widely used source for the prediction of potential snail habitats [26], [27], [30], [34], [35], but their high cost has limited broad usage of this source for general users, especially for the routine monitoring with respect to disease control. However, the China-Brazil earth resources satellite (CBERS), which provides 20 m resolution images, is completely free of charge for researchers, providing a good opportunity to explore its value for the detection of snail habitats instead of costly ETM images.

The normalized difference water index (NDWI), put forward as a specific means to extract water information, proved to be an efficient index for predicting aquatic regions [39]. Since few studies have reported this application, it was felt to be worthwhile to discuss the feasibility of applying NDWI (as well as NDVI) in the process of identifying snail habitats. We explored the use of 20 m multi-temporal CBERS images to identify potential snail habitats with the simultaneous application of NDWI and NDVI as it would solve the above-mentioned issues. Some ideas, found useful for detecting and monitoring snail habitats, are provided together with the results with the expectation to shed some light on future effective monitoring of snail habitats.

## Materials and Methods

### Study Area

**Figure 1:**
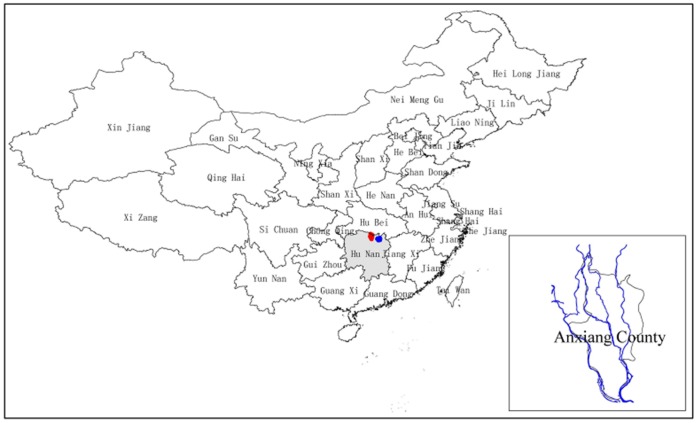
A map illustrating the location of Anxiang County and the positions of the major river systems. The polygon in gray is Hunan Province; the red and the blue parts in the polygon are the positions of Anxiang County and Dongting Lake, respectively. The enlarged map in the right-bottom corner shows the positions of the major river systems in blue in Anxiang County.

Anxiang County (Figure 1), situated in the northwest of Dongting Lake in Hunan province, is a typical *S. japonicum* endemic area in the lake and marshland regions of China. It covers an area of approximately 1,087 km^2^, comprises 263 villages and has an at-risk population of about 429,000 people. Four major river systems (Lishui, Songzi, Hudu, and Ouchi) and the humid subtropical monsoonal climate, with an average annual temperature about 16.4°C and average rainfall of about 1,130 mm, provide an ideal environment for *O. hupensis*.

### Base Map

Digitized polygon maps of Anxiang County and the major rivers at a scale of 1∶250,000 were obtained from local government as base for related data/results for visual interpretation.

### RS Satellite Images

RS allows the observation of objects, surfaces or phenomenon from a long distance without actual physical contact [40], [41]. Since it is a technology with strong governmental investment supporting users from many different areas, it is a service that has become relatively inexpensive. It is also a rapid way of acquiring up-to-date information with a wide regional coverage. Indeed, it is the only practical approach to rapidly gather information about areas where it is not possible to carry out ground surveys (inaccessible regions like flooded areas, mountainous areas and foreign areas). RS also makes it feasible to construct base maps in the absence of detailed land surveys, enabling the continuous acquisition of data over time as well as space [40], [41]. Therefore, RS provides an ideal tool for mapping, predicting and monitoring disease trends, including the dynamics of vectors and intermediate hosts. CBERS is a joint program developed by China and Brazil since 1988, successfully launching its CBERS-2 satellite on 21 October 2003. This satellite has five spectral bands with 20 m spatial resolution and a 120 km swath width on the ground. In addition to the panchromatic band 5 (0.51–0.73 µm), bands 1–4 absorbs blue (0.45–0.52 µm), green (0.52–0.59 µm), red (0.63–0.69 µm) and near infrared (0.77–0.89 µm) light, respectively. This is similar to what the Landsat ETM instrument provides; hence they were comparable in the visible (bands 1–3) and the near infrared bands (band 4) (see previous reports for detailed information [42]). CBERS images and measurements are freely available for academic users in China. Because of the similarities between the Landsat ETM bands and those of CBERS, many previous ideas of studying vector-borne diseases using ETM images can be adapted and used with CBERS images without difficulty. To date, however, no studies have reported such applications.

In this study, six bimonthly consecutive CBERS images with a spatial resolution of 20 m, covering Anxiang County, were obtained free of charge from the CBERS website (http://www.cresda.com/n16/index.html ). They were taken on 20 December 2003, 10 February 2004, 2 April 2004, 19 June 2004, 10 August 2004, and 27 October 2004, respectively.

### Data Preprocessing

The image of 20 December 2003 was selected as reference layer and over 20 clearly distinguishable and known coordinate locations (e.g., road crossings, intersection points of rivers) were designated as ground control points (GCPs). The other five images were georeferenced to this layer using the selected GCPs. Polynomial transformation for geometric modeling and bilinear interpolation for the resampling were used. The final root mean square (RMS) errors in the process of georeferencing images were set at less than 0.5 pixels.

### Identification of Snail Habitats

NDWI was derived from six CBERS images in order to identify the water regions using similar principles as those used for NDVI. It is calculated as [39]:
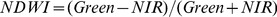
(1)where, *Green* represents the band that encompasses the reflected green light and *NIR* indicates the band that reflected the near-infrared radiation, corresponding to CBERS Band 2 and Band 4, respectively. NDWI maximizes the reflectance of water features by using green light wavelengths instead of the near-infrared radiation, thus minimizing the low reflectance of NIR by water features, while taking advantage of the high reflectance of NIR by terrestrial vegetation and soil features, making it is a good index for identifying aquatic areas. Just as NDVI, the range of NDWI is (−1, 1) with water surfaces resulting in relatively strong positive values, while other surface features are lower or negative. To determine the appropriate threshold for NDWI measurement, we first selected 50 points in regions characterized by water features and extracted their NDWI values in each image. Based on the distribution of the NDWI and the perspective of small probability event, the lower limit of 95% confidence interval was calculated as the threshold of NDWI to differentiate the water regions (>threshold) from other features. Then, the extracted aquatic regions in each image were converted to polygons and the surface areas of these water polygons were calculated accordingly. The images with the maximum and minimum area of aquatic regions were used to represent the wet season and the dry season, respectively. The differences in these images with respect to water were obtained through the subtraction algorithm to represent the areas that could be characterized as “land in winter – water in summer”.

Following this, NDVI was calculated for each image using the formula:
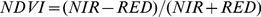
(2)where, *RED* stands for the spectral reflectance measurements acquired in the visible red region and NIR is the near infrared (as used in the NDWI formula). NDVI is a widely used index to identify the regions with vegetation coverage. Fifty snail habitats were obtained from historical records and then the NDVI values in the dry season were extracted for these 50 snail habitats. The lower limit of the 95% confidence interval was used as threshold to obtain the areas with vegetation coverage, i.e. the “no grass– no snails” areas (>threshold).

To discriminate the vegetation types suitable for snail survival from other unfavorable vegetation types, the idea of the patterns of vegetation growth was used. For the time-series images, the standard deviation (SD) of NDVI values in each grid was computed to represent the variance of vegetation. Then, the NDVI’s SD values in the 50 true snail habitats were extracted and the lower limit of the 95% confidence interval for the 50 SDs was calculated as the threshold to select the regions where the pattern of vegetation growth was appropriate for snail survival (>threshold).

Finally, the three regions termed “land in winter – water in summer”, “no grass – no snails” and “appropriate pattern of vegetation growth”, respectively, were overlaid. The overlapping areas obtained were taken to indicate the potential snail habitats in Anxiang County.

**Figure 2:**
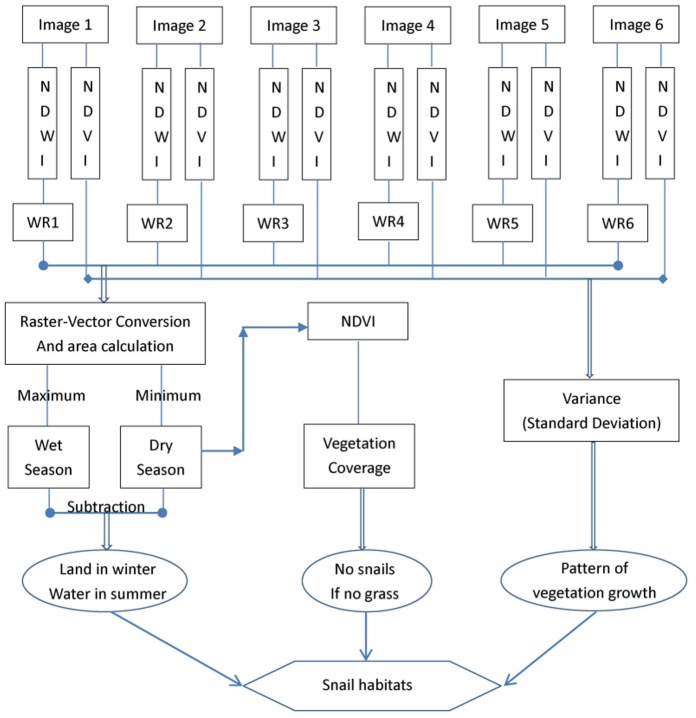
Schematic flowchart for identifying the snail habitats in Anxiang County, displaying the general process for detecting snail habitats by combining three results of “land in winter - water in summer”, “no snails - no grass”, and “pattern of vegetation growth (suitable for snail survival)”. (WR means *water region*).

The flowchart for the above data processing and analysis is displayed in Figure 2. All these steps were accomplished in ERDAS IMAGINE 9.2 (Leica Geosystems Geospatial Imaging, LLC, Norcross, GA, USA) and ArcGIS 10 software (Environmental Systems Research Institute (ESRI),Inc., Redlands, CA, USA).

### Model Validation

We randomly selected 200 points in Anxiang County to evaluate the results of localized snail habitats. From the historical records and field investigations of snail habitats in local regions, we determined whether each selected point was located in the snail habitats and then compared the result with those extracted from the model to judge whether they were correctly classified. The sensitivity and specificity of the suggested approach were calculated.

## Results

NDWI values from water regions are summarized in Table 1. Except for 2 April 2004, the minimum NDWI values in the CBERS images are all positive with corresponding NDWI thresholds are higher than 0.1.

**Table 1:** Summaries of NDWI values in water points in six images.

Date	Min	Q1	Mean	Q3	Max	Std	Threshold
20 December 2003	0.24	0.30	0.32	0.35	0.41	0.04	0.26
10 February 2004	0.24	0.28	0.31	0.33	0.41	0.04	0.24
2 April 2004	−0.02	0.05	0.07	0.10	0.13	0.04	0.00*
19 June 2004	0.05	0.17	0.19	0.22	0.29	0.05	0.11
10 August 2004	0.13	0.16	0.18	0.19	0.25	0.03	0.13
27 October 2004	0.21	0.23	0.26	0.28	0.31	0.03	0.21

* the exact value is 0.0044; Q1 and Q3 represent the first and third quartiles, respectively.

**Figure 3:**
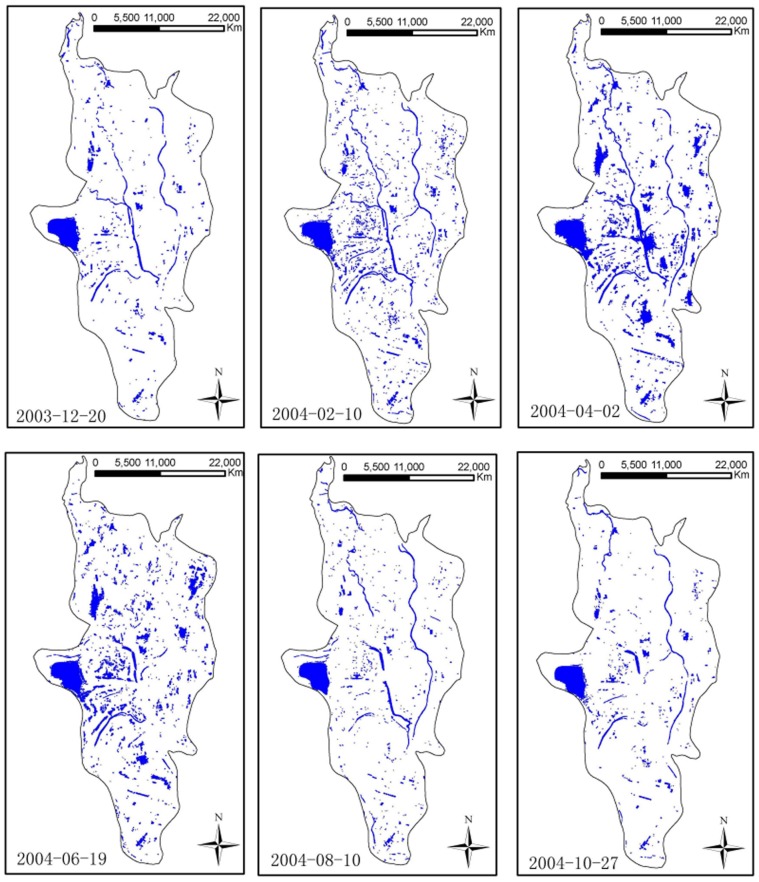
Distribution of water regions in different seasons extracted by the index of NDWI. The areas of aquatic regions from the images on 2 April 2004 and 27 October 2004 were the maximum and the minimum, respectively.

**Figure 4:**
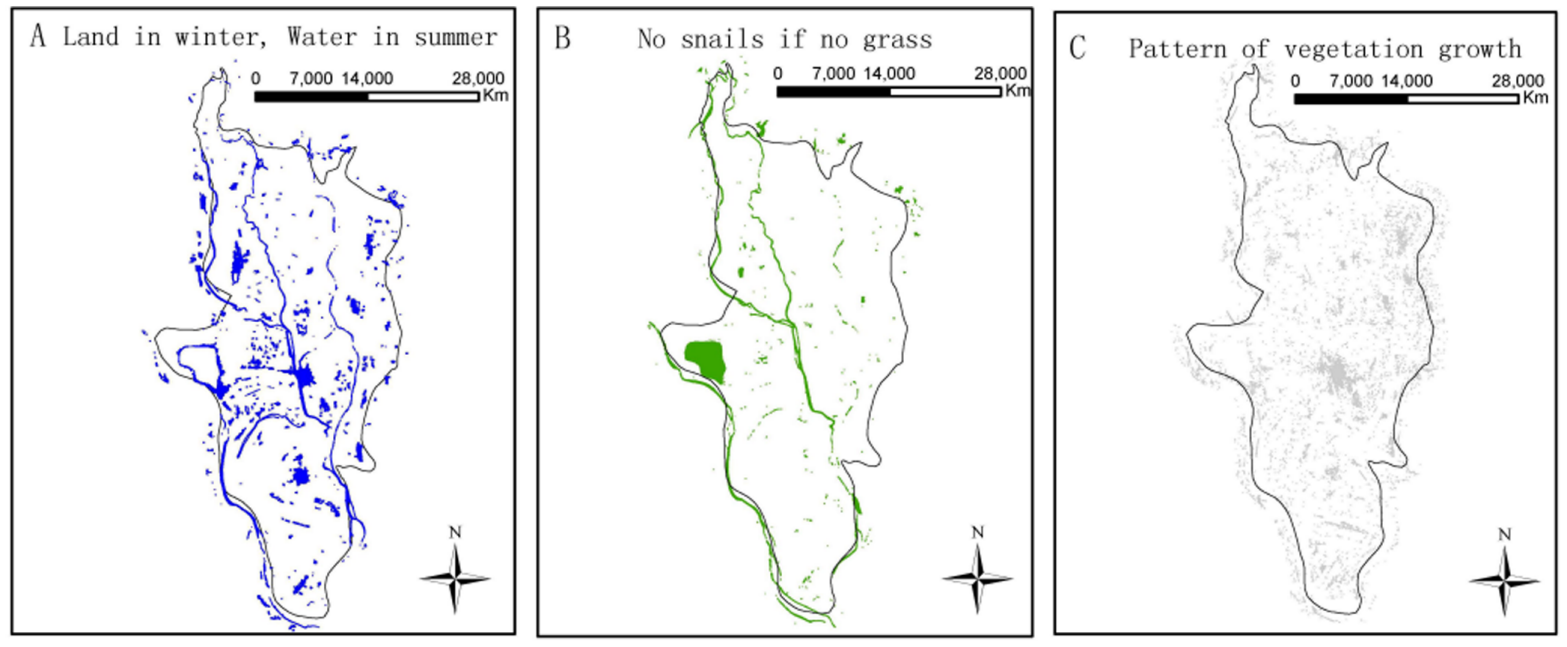
Distributions of extracted regions based on different methods. (A)The regions of “land in winter - water in summer” obtained by subtraction of water season and dry season; (B) the regions of “no snails - no grass”, i.e. the regions with vegetation coverage; and (C) the vegetation regions suitable for snail survival discriminated by the variance of vegetation growth.

Figure 3 depicts the extracted water regions from six CBERS images. The aquatic areas totaled 36.07 km^2^ on 20 December 2003, 41.23 km^2^ on 10 February 2004, 52.04 km^2^ on 2 April 2004, 39.67 km^2^ on 19 June 2004, 33.11 km^2^ on 10 August 2004, and 32.38 km^2^ on 27 October 2004. Thus, the image from 2 April 2004 was chosen to represent the wet season and 27 October 2004 to represent the dry season. The difference between the water regions extracted from the wet and the dry seasons represents the regions of “land in winter – water in summer” displayed in Figure 4 (A).

**Figure 5:**
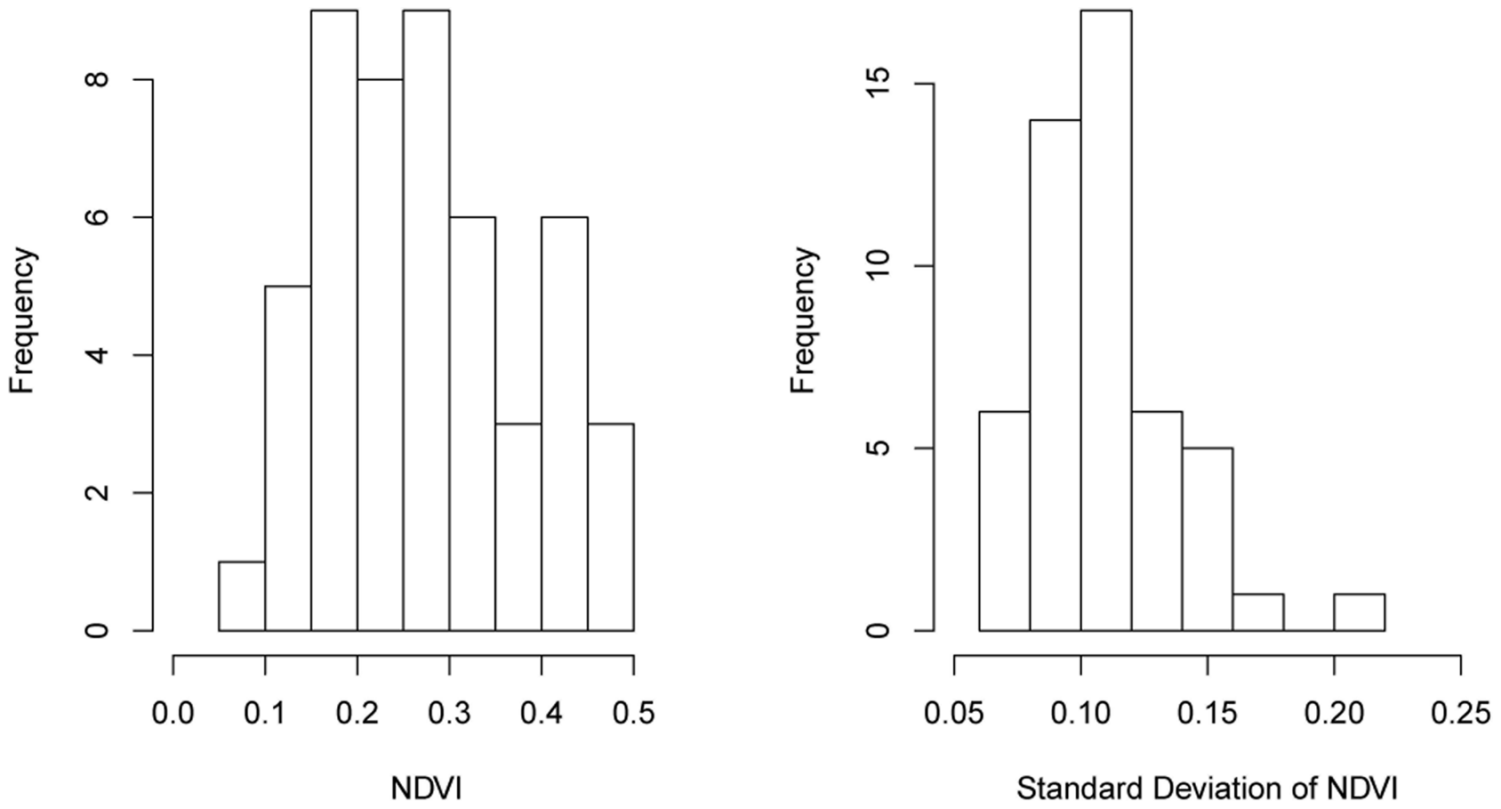
Distributions of NDVI values of the true snail habitats in the dry season and the variance of the true snail habitats in different seasons.

The NDVI values from true snail habitats are shown in Figure 5 (left); their mean and SD are 0.27 and 0.11, respectively. The threshold used to detect the regions with vegetation coverage was 0.09, and the vegetation regions indicating the “no snails – no grass” are displayed in Figure 4 B.

The threshold for identifying the pattern of vegetation growth suitable for snail survival is 0.06 (Figure 5 right) and the recognized regions depicted in Figure 4 C.

**Figure 6:**
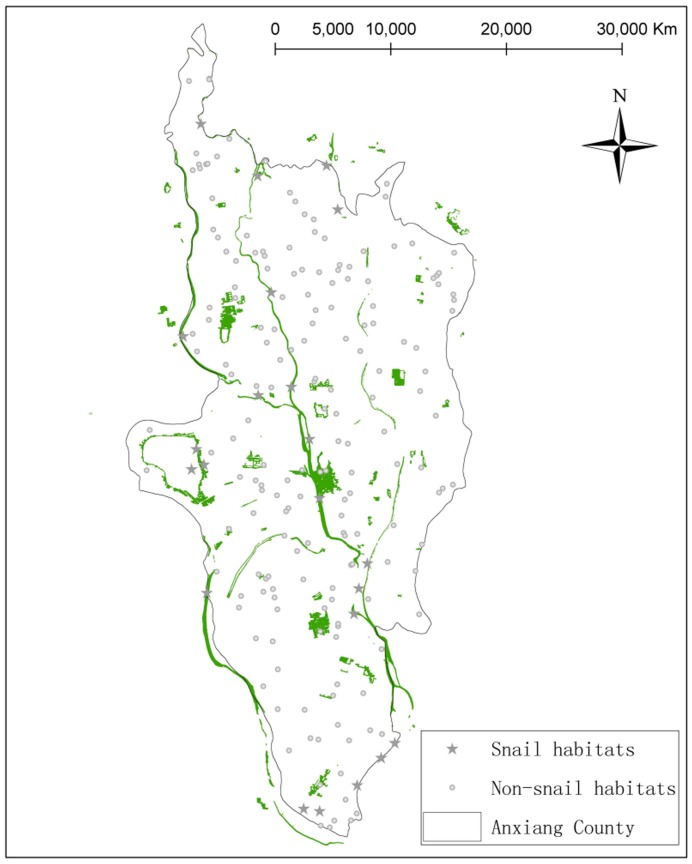
Distributions of snail habitats and the validation points. The regions depicted as green are the snail habitats identified by our approach. The small circles in gray indicate the environments without snails and the stars in gray represents the habitats with live snail.

Figure 6 displays the localizations of the snail habitats obtained by overlaying the three feature layers (Figure 4), most of which were found to be distributed along the river systems. The sensitivity and specificity of the suggested approach were 63.64% (14/22) and 78.09% (139/178), respectively.

## Discussion

Based on CBERS images, this study used two environmental features (namely water and vegetation) to identify the snail habitats, which have previously proved useful.

NDWI has been used to identify aquatic regions [39]. However, the NDWI thresholds for extracting these regions are different for images from different seasons, suggesting that the methods used in previous studies to choose this value subjectively were not rational. An objective approach, resorting to statistical index, should be a feasible way to avoid this issue. The NDWI threshold on 2 April 2004 is negative because the provided CBRES images did not perform the radiance transformation. This was not an error, but it supports the idea that it is right to decide the threshold objectively in practical applications for identifying the aquatic regions. The grid values in RS images provided are not always standard data and most of the users are not RS experts, so choosing a threshold subjectively may well lead to biased or even incorrect results. Besides, previous studies suggest that the choice of the two images representing the wet and the dry season, respectively, were also subjective. For example, Guo et al. used April and August for this purpose in their Poyang Lake study [26], while Li et al chose December and June [43]. Applying NDWI to the time-series RS images provides an objective way to select the images for the wet and the dry season just as done in this study. Also, compared to previously used classification methods, NDWI is easier to use and has the same advantages as NDVI. For these reasons, it is very probable that NDWI will become an index as popular as NDVI for the identification of snail habitats in the field of schistosomiasis research and control.

NDVI has been widely used for the prediction of potential snail habitats through quantifying regions of green vegetation [26], [27], [35], but the threshold used by different research groups were different [34]. The NDVI threshold in this study, determined to be 0.09, is slightly lower than previous reports. The applied RS images are different and the spatial resolutions are also slightly different, possibly accounting for this minor difference. Besides, previous studies determined those values subjectively, while the threshold may vary in RS images from different seasons because the quality of RS images can be affected by various factors such as cloud shadows [40], [41]. Therefore, as discussed above, the objective approach of using a statistical index to decide the NDVI threshold is recommended.

In this paper, we did not only rely on the NDVI but used also a new index representing the pattern of vegetation growth to further discriminate the vegetation types suitable for snail survival from those that are not. The calculated threshold is 0.06, which prompts the notion that the vegetation in the regions of snail habitats does undergo changes. This is intuitively correct because the vegetation has its own growth cycle: sprouting, growing, maturing, and then wilting. In the field surveys, we do find the phenomenon that snail-supporting vegetation (e.g., Carex) shows larger variances than unsuitable vegetation (e.g., weeds) [13]. This is the first report of using this threshold to facilitate the identification of snail habitats and more studies from different regions are needed to gain experience. We strongly recommend that other researchers explore this approach in future studies aimed at identifying snail habitats or similar studies.

The identified snail habitats are mainly distributed along rivers, suggesting that the presence of water is important, which is in accordance with previously published reports [7], [27]. According to our evaluation, we found that the sensitivity and specificity of our approach were 63.64% and 78.09%, respectively. That is lower compared to that reported previously, which may have overstated the results of model assessment. Some assessments were performed only in areas predicted to be snail habitats, but not in regions predicted to be free from snails [26], [27], while other evaluations did not adhere to the rule of randomization, that is, their snail and non-snail habitat sites were not randomly sampled [30], [34], [44]. Besides the high cost of RS images, overstated accuracy of RS-based model prediction may be one of the reasons that RS techniques have not become as effective a tool for schistosomiasis monitoring as it could be. The free availability of CBERS images, however, makes it possible that this will become the routine tool for monitoring the distribution and dynamics of snail habitats. More research into improving the model’s accuracy is, however, needed.

Finally, we should point out that although the presence of vegetation and water is an important and necessary condition for snails and snail habitats, many other factors are also needed. Auxiliary thematic data dealing with soil, altitude, hydrological conditions, for example, would improve the sensitivity and specificity when monitoring snail habitats or predicting their presence. Further study on how to effectively integrate these features needs consideration [30], [44]. Besides, just as previous (un)supervised classification, our approach also produces a result of “truth” (conditions satisfied) or “false” (conditions not satisfied) for each grid in the RS images. This may be too arbitrary. Some new approach, e.g. as fuzzy classification can produce a dataset, which no longer results in either “Yes” or “No”, but rather as a fuzzy continuous set of values ranging from 0 (False) to 1 (True) in an ambiguous manner [30], [45], [46]. This kind of result should be more meaningful and could be another interesting research direction in the future.

In conclusion, we applied the two environmental features of water and vegetation extracted from the multi-temporal CBERS images to identify snail habitats. NDWI was first applied to locate the water regions and the pattern of vegetation growth to differentiate the vegetation suitable for snails from that unsuitable for snail survival was then explored through joint application with NDVI. The model, based on CBERS images available free of charge, holds promise for the future monitoring of snail habitats and predicting of the distribution and dynamics of snails in schistosome-affected regions that lack accurate surveillance capabilities. This approach is gaining credence in the face of local ecological transformation caused by various factors such as the potential of climate change and the construction of hydraulic projects. Indeed, it could prove to be one of the most important tools for the ongoing national schistosomiasis control program. However, more research to improve the model’s accuracy is urgently needed.
